# Fast-Track Protocol for Carotid Surgery

**DOI:** 10.3390/jcm14124294

**Published:** 2025-06-17

**Authors:** Noemi Baronetto, Stefano Brizzi, Arianna Pignataro, Fulvio Nisi, Enrico Giustiniano, David Barillà, Efrem Civilini

**Affiliations:** 1 Vascular Surgery Unit, IRCCS Humanitas Research Hospital, Via Manzoni 56, 20089 Rozzano, Milan, Italy; noemi.baronetto@humanitas.it (N.B.); arianna.pignataro@humanitas.it (A.P.); david.barilla@hunimed.eu (D.B.); efrem.civilini@hunimed.eu (E.C.); 2 Department of Medicine and Surgery, University of Insubria, 21100 Varese, Italy; 3 Department of Anesthesia and Intensive Care Units, IRCCS Humanitas Research Hospital, 20089 Rozzano, Milan, Italy; fulvio.nisi@humanitas.it (F.N.); enrico.giustiniano@humanitas.it (E.G.); 4 Department of Biomedical Sciences, Humanitas University, Via Rita Levi Montalcini 4, 20072 Pieve Emanuele, Milan, Italy

**Keywords:** fast-track protocol, carotid endarterectomy, enhanced recovery, eversion technique, local anesthesia

## Abstract

**Background/Objectives:** Fast-track (FT) protocols have been developed to reduce the surgical burden and enhance recovery, but they still need to be established for carotid endarterectomy (CEA). In this scenario, carotid stenting has gained momentum by answering the need for a less invasive treatment, despite a still debated clinical advantage. We aim to propose a FT protocol for CEA and to analyze its clinical outcomes. **Methods**: This retrospective, monocentric study enrolled consecutive patients who underwent CEA for asymptomatic carotid stenosis using an FT protocol between January 2016 and December 2024. Patients undergoing CEA for symptomatic carotid stenosis, carotid bypass procedures, and combined interventions were excluded. Our FT protocol comprises same-day hospital admission, exclusive use of local anesthesia, non-invasive assessment of cardiac and neurological status, and selective utilization of cervical drainage. Discharge criteria were goal-directed and included the absence of pain, electrocardiographic abnormalities, hemodynamic instability, neck hematoma, or cranial nerve injury, with a structured plan for rapid readmission if required. Postoperative pain was assessed using the numerical rating scale (NRS), administered to all patients. The perioperative clinical impact of the protocol was evaluated based on complication rates, pain control, length of hospital stay, and early readmission rates. **Results**: Among 1051 patients who underwent CEA, 853 met the inclusion criteria. General anesthesia was required in 17 cases (2%), while a cervical drain was placed in 83 patients (10%). The eversion technique was employed in 765 cases (90%). Postoperative intensive care unit (ICU) monitoring was necessary for 7 patients (1%). The mean length of hospital stay was 1.17 days. Postoperatively, 17 patients (2%) required surgical revision. Minor stroke occurred in three patients (0.4%), and acute myocardial infarction requiring angioplasty in two patients (0.2%). Inadequate postoperative pain control (NRS > 4) was reported by five patients (0.6%). Hospital readmission was required for one patient due to a neck hematoma. **Conclusions**: The reported fast-track protocol for elective carotid surgery was associated with a low rate of postoperative complications. These findings support its clinical value and highlight the need for further validation through controlled comparative studies. Furthermore, the implementation of fast-track protocols in carotid surgery should prompt comparative medico-economic research.

## 1. Introduction

The Fast-track (FT) concept, introduced in the 1990s, represents a multimodal approach aimed at reducing patient morbidity and mortality after surgery [1]. By integrating multiple evidence-based perioperative interventions, FT protocols are designed to mitigate surgical stress, minimize organ dysfunction, and accelerate postoperative recovery [1].

These protocols, founded on the fundamental principles of safety and efficiency, have been successfully implemented across various surgical specialties [2,3,4,5,6,7].

In the field of vascular surgery, the comprehensive adoption of FT protocols is still evolving [7,8,9,10,11,12,13,14]; one specific area requiring further development is carotid endarterectomy (CEA). In this scenario, the ongoing evolution of endovascular procedures, which are increasingly less invasive, innovative, and associated with improved efficacy and safety, has significantly influenced treatment paradigms. Carotid artery stenting (CAS) has gained recognition as a viable alternative to CEA, particularly in patient populations where traditional open surgery is perceived as higher risk, such as the elderly or those with significant comorbidities [15,16]. However, fast-track surgical protocols have demonstrated their applicability even in high-risk patient groups, including octogenarians and those with multiple comorbidities, who might traditionally be steered toward endovascular options. These protocols offer a safe and effective pathway, with notable advantages such as shorter hospital stays, reduced healthcare costs, and robust procedural safety.

The aim of the study is to analyze clinical outcomes of our comprehensive FT protocol in order to assess its potential to enhance patient recovery.

## 2. Materials and Methods

This retrospective, single-center study evaluated the outcomes of our fast-track protocol for elective CEA at the Vascular Surgery Department of Humanitas Research Hospital, Milan, over an eight-year period.

The FT protocol for carotid surgery was introduced in 2015, marking the beginning of its clinical implementation. A one-year learning curve was essential for achieving full integration, during which the protocol was systematically introduced and refined. This initial phase involved evidence-based analysis and multidisciplinary discussions, engaging nurses and hospital management teams to ensure a structured and effective adoption.

Patients with symptomatic carotid stenosis, patients undergoing carotid artery stenting, and patients undergoing combined surgical procedures were excluded from the study.

Patients recommended for CAS instead of CEA included those who were not deemed suitable for open surgery during the pre-admission assessment, as well as those with carotid restenosis.

Indications for CEA were established in accordance with the latest European Society for Vascular Surgery (ESVS) guidelines.

Since our study focused exclusively on asymptomatic patients, patient selection was almost entirely based on dual-operator DUS strategy with validated inter-observer consistency. Furthermore, the indication for surgery was given in the presence of one or more imaging or clinical characteristics that may be associated with an increased risk of late stroke, provided 30-day stroke/death rates are <3%. 

According to the current European guidelines, the use of two independent duplex ultrasound (DUS) examinations performed by different operators is considered an acceptable alternative to preoperative CTA in selected cases. Our institutional protocol adheres to this recommendation, employing a dual-operator DUS strategy with validated inter-observer consistency, in asymptomatic patients. We therefore reserved CTA for patients with inconclusive or discordant duplex ultrasound findings, prior imaging from other centers, or specific anatomical considerations.

As for the risk of missing tandem lesions without performing CTA, the literature does not give a clear indication when speaking of asymptomatic patients. Furthermore, we routinely perform intraoperative angiography, which allows us to detect intracranial lesions, if any.

All patients enrolled in our study were already on the best medical therapy at the time of surgical evaluation, including at least one antiplatelet agent and statins [17].

Over the nine-year study period, we observed a progressive shift in clinical practice toward a more conservative management of asymptomatic carotid stenosis, driven in part by the growing effectiveness of pharmacologic therapies. As a result, the threshold for offering CEA has gradually increased, and the frequency of surgical indication has declined. For instance, patients with moderate (e.g., 60%) asymptomatic stenosis and no imaging features of plaque instability are no longer routinely offered surgical treatment in our institution.

Once the diagnosis is confirmed, patients with asymptomatic carotid stenosis are placed on an elective surgical list and evaluated during a dedicated pre-admission visit involving both anesthesiology and cardiology teams. Broader multidisciplinary discussions—including neurologists—are generally reserved for patients with symptomatic carotid disease or borderline indications, where additional diagnostic workup and more complex therapeutic considerations are warranted. This tiered approach ensures appropriate resource allocation while maintaining individualized, evidence-based care planning.

From January 2016 to December 2024, at the Vascular Surgery Department of Humanitas Research Hospital (Milan), clinical data about 1051 consecutive patients undergoing elective CEA were collected and retrospectively analyzed.

The details of our protocol are explained in Appendix A and listed in Table 1.

The primary endpoint was to assess the impact of the fast-track protocol on postoperative rate of major complications (death, occurrence of major stroke, minor stroke, and acute myocardial infarction).

The secondary endpoints were to evaluate the patient’s mean operative time, intensive care unit (ICU) admission, hemodynamic stability, pain control, reinterventions, mean length of hospital stay, 30-day readmission rate. Pain was considered significant if it was graded 4 or higher according to the numerical rating scale (NRS). We considered blood pressure control satisfactory if intraoperatively the systolic arterial pressure (SAP) remained above 140 mmHg and less than 200 mmHg during the clamping phase and its variation was <20% compared to basal SAP level (measured at the beginning of surgery). Postoperatively, adequate pressure control was defined as SAP < 140 mmHg. Potential sources of bias are the relatively small sample size and the retrospective monocentric nature of the study.

### Statistical Methods

Descriptive statistics were used to summarize patient demographics, clinical characteristics, and perioperative outcomes. Categorical variables, such as complication rates (e.g., major stroke, minor stroke, myocardial infarction), the use of general anesthesia, and the need for cervical drainage, were expressed as frequencies and percentages. Continuous variables, including the length of hospital stay and pain scores were presented as means with standard deviations or ranges.

Comparisons with published literature were made qualitatively to contextualize the findings and evaluate the clinical impact of our FT protocol. No formal inferential statistical tests were conducted for inter-study comparisons due to the heterogeneity of external data.

The primary endpoint and secondary endpoints (e.g., ICU admission, postoperative pain scores, and reintervention rates) were analyzed to assess the efficacy of the fast-track protocol. Missing data were addressed by excluding cases with incomplete records for specific variables, though this constituted a negligible portion of the dataset. Given the retrospective design, potential selection biases and confounders were acknowledged but not explicitly controlled through multivariable analysis or matching techniques.

Although sensitivity analyses were not conducted, the findings are consistent with the aim of evaluating the feasibility and safety of the fast-track protocol for CEA, in comparison to established benchmarks in the literature.

## 3. Results

Out of the 1051 patients initially assessed, 853 met the inclusion criteria and were included in the study. All the reported outcomes refer to the immediate post-operative period.

At baseline, the contralateral carotid artery was found to be occluded in 22 patients and near-occluded in 5; 103 patients had previously undergone carotid intervention (either endarterectomy or stenting), while 33 exhibited a hemodynamically significant stenosis.

The demographic characteristics of these patients are detailed in Table 2.

All patients underwent surgery based on duplex ultrasound (DUS) evaluations performed by two different operators, except for 51 (6%) of patients, who underwent computed tomography angiography (CTA) of the supra-aortic trunks. CTA was obtained in case of discrepancies between the two duplex ultrasound reports, when the ultrasound findings were inconclusive, or when patients had previously undergone CTA based on an external indication. Among the 853 patients, 7 (0.6%) required conversion from LA to GA: 4 due to poor compliance during the procedure and 3 due to local anesthesia-related toxicity. Adequate intraoperative blood pressure control was obtained in 76.6% of patients. An intraoperative shunt was indicated in 84 cases (10%) due to the onset of neurological deficits following carotid clamping. In total, 765 patients (90%) underwent eversion CEA, while in 88 cases (10%), a longitudinal arteriotomy was performed with Dacron patch closure. Cervical drainage was inserted in 83 patients (10%). The average operative time was 83 min. Postoperative monitoring in the ICU was indicated for seven patients (0.8%), and the average hospital length of stay was 1.17 days. During the hospital stay, from a neurological standpoint, we recorded three cases (0.36%) of minor stroke and zero cases of major stroke. Cardiovascular complications included two cases (0.2%) of acute myocardial infarction both of which required subsequent angioplasty. Surgical revision for lateral neck hematoma was necessary in 17 patients (2%), either on postoperative day 0 or 1. No deaths occurred in the study cohort. One patient (0.2%) was readmitted 1 week postoperatively for surgical revision of a late-onset lateral neck hematoma. Transient cranial nerve damage was observed in nine patients (1%). The patients who developed hypertension (systolic blood pressure > 160 mmHg) in the postoperative period were 316 (37%). Regarding pain management, only 5 patients (0.6%) reported poor pain control (NRS > 4).

## 4. Discussion

This retrospective, single-center study evaluated the outcomes of an FT protocol for elective CEA. This protocol is a multimodal evidence-based approach to surgical care that begins in the preoperative setting and extends through to patient discharge in the postoperative period. This goal-directed pathway involves a multidisciplinary team to reduce complications, accelerate recovery, and shorten hospital stays. In the realm of aortic surgery, a body of literature delves into the applicability of fast-track programs [2,3,4,5,6,7,8,14]. However, applying such protocols to carotid surgery presents unique challenges that significantly limit their feasibility due to their high risk of developing hyperacute life threatening conditions [1]. One major hurdle is hemodynamic instability, particularly postoperative hypertension. This condition frequently follows CEA, especially with the eversion technique, and is attributed to baroreceptor dysfunction. While typically transient, hypertension often peaks within the first few postoperative hours and increases the risk of severe complications, including cervical hematoma, myocardial ischemia, and, in some cases, cerebral hyperperfusion syndrome [18,19]. These risks necessitate close hemodynamic monitoring, complicating efforts to streamline postoperative care. Another critical issue is the risk of airway obstruction, stemming from factors such as traumatic mucosal edema, tracheal compression by hematomas, or venous and lymphatic congestion. To mitigate this, lateral cervical drains are often placed to prevent tracheal compression, but their use can delay discharge and elevate the risk of postoperative infections, further complicating the implementation of fast-track protocols [20]. Additionally, the surgical field’s proximity to critical structures increases the likelihood of cranial nerve injury. Although most injuries result in transient nerve dysfunction rather than permanent damage, the potential for complications underscores the delicate and intricate nature of carotid surgery [21]. Specifically addressing all those major criticisms, we have developed an FT protocol applicable in carotid surgery aimed at making the surgical procedure as minimally stressful as possible for the patient.

Patient selection for treatment is primarily based on non-invasive diagnostics, with carotid stenosis > 70% [17] confirmed through two separate DUS evaluations conducted by different operators. CT angiography was selectively performed in 51 patients (6%) where DUS findings were inconclusive/divergent between the two operators, or when specific anatomical challenges existed, such as hostile neck anatomy, obesity, a high carotid bifurcation, or heavily calcified plaques that could not be adequately assessed via DUS. In asymptomatic patients, preoperative brain CT scans are not routinely performed. This approach helps to significantly reduce the waiting time between diagnosis and surgical intervention, as DUS examinations have a much shorter scheduling delay compared to CT angiography or brain CT. Not having to perform a second-level examination allows for organizing the pre-admission process for patients in a single day, thus reducing the waiting times for surgery, which at our center are under 30 days.

**Figure 1:**
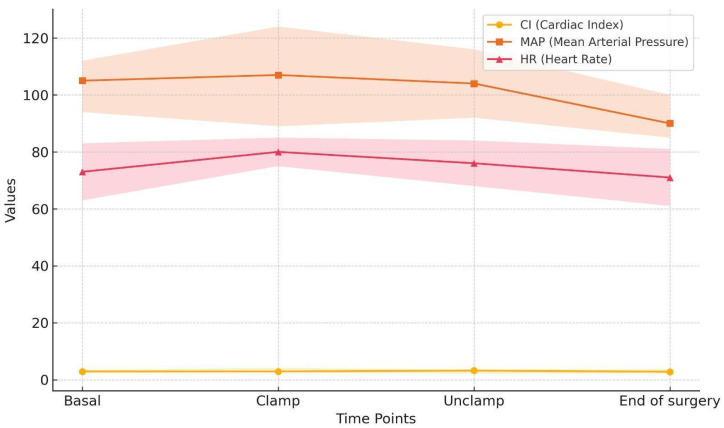
Intraoperative hemodynamic trends.

Most of our patients (60%) presented in the operating room with systolic blood pressure values exceeding 160 mmHg, which might be related to withholding of antihypertensive medications. However, this phenomenon facilitated avoidance of intraoperative hypotension and the achievement of adequate blood pressure control during carotid artery clamping in 76.6% of the patients. Cardiac output on the other hand was stable throughout the surgery, with values exceeding 2.5 L/min/m^2^, thus ensuring stable organ perfusion pressure (Figure 1).

In the postoperative period, 316 patients (37%) experienced at least one hypertensive peak, for which, according to the protocol, an α2-adrenergic receptor agonist was administered. No hypertensive emergencies or urgencies were recorded.

Intraoperatively, the FT protocol specifies that the procedure is performed under local anesthesia (LA) [22,23,24] [Appendix A].

Among the 853 patients included in the study, 836 (98%) successfully underwent the procedure under LA. Conversion to general anesthesia (GA) was required in seven patients (0.8%): three cases (0.3%) due to local anesthetic toxicity and four cases (0.4%) due to poor compliance. In our experience, the use of LA has proven to be applicable across a wide range of patients. The low rate of anesthetic toxicity occurred during the early phases of implementing the fast-track protocol and were attributed to accidental intravascular injection, reflecting the surgical team’s learning curve. At the same time, the low incidence of toxicity highlights the reproducibility and robustness of the technique, demonstrating its capacity to be consistently and safely applied across different clinical settings as expertise develops. Both patients were safely converted to GA with no lasting effects. Patients demonstrating poor compliance under LA represented a minimal portion of the cohort. The ESVS 2023 guidelines [17] emphasize that the choice of anesthesia, whether locoregional or general, should be left to the discretion of the surgeon or anesthesiologist performing the procedure, considering factors such as local expertise, patient preferences, and the antiplatelet strategy employed. At our center, these decisions are not made unilaterally by the surgical or anesthesiology team; they are always discussed in a multidisciplinary setting considering the patient’s comorbidities, risk factors, and age. This collaborative approach ensures that all perspectives are considered, allowing for the selection of the safest and most appropriate treatment for each patient. Based on the evidence provided by Gomes et al., LA is likely to be the preferred approach for carotid endarterectomy in patients for whom either anesthetic option is clinically appropriate [24].

Performing the procedure under LA enables direct clinical neurological monitoring, ensuring the patient maintains stable consciousness and no neurological deficits arise after carotid clamping. This approach allows for selective shunting only in patients who exhibit post-clamping central neurological deficits, which, in our experience, is very low and accounts for approximately 10% of cases (84 patients). By avoiding routine shunt use, this strategy minimizes the associated risks and complications, such as arterial dissection, thrombosis, and embolization [25].

Our protocol specifies that carotid endarterectomy, when shunt placement is not required, is performed using the eversion technique. This approach offers several advantages for experienced surgical teams: it shortens the operative time, eliminates the need for a patch (reducing the risk of infection), and decreases costs associated with surgical materials.

Wound drainage was selectively employed in patients on dual antiplatelet therapy (DAPT) and in patients in whom hemostasis has not been achieved within 10 min, comprising 10% of the study population. In our cohort, only 17 patients (<2%) developed lateral neck hematoma requiring surgical revision, all of which were performed on postoperative day 0 or 1. In our experience, the use of drainage in the context of acute bleeding is generally of limited value, as it does not significantly reduce the rate of reintervention or demonstrate to be of any help in case of acute arterial bleeding. Selective drainage not only supports shorter hospital stays but also improves postoperative pain control, facilitates the patient’s mobility and reduces the risk of infection. Notably, no cases of infection were observed in our case series.

The selective use of cervical drains in our study contrasts with other approaches advocating routine drain placement [20,26]. To date, there are few published studies evaluating the “to drain versus not to drain” approach. Recently, a meta-analysis [19] has demonstrated that routine drain placement is not necessary following CEA and does not prevent neck hematoma. Our study supports the current European clinical practice guidelines recommendation on selective drain placement [17].

At the end of the surgical procedure, patients undergo close monitoring in the operating room’s recovery for 1 h. Following this period, if clinical conditions and vital signs fall within normal ranges, patients are readmitted back to the ward [Appendix A]. In contrast to the guidelines, where it is stated that most patients should be transferred to a vascular ward after the initial 24 h monitoring period, with non-invasive blood pressure and neurological monitoring, our approach has demonstrated that most patients can be safely managed without the need for intensive monitoring, minimizing the need for ICU admissions. Specifically, in our series, only 0.8% of patients required postoperative monitoring in the ICU, which highlights the effectiveness and safety of our protocol in reducing the need for high-dependency care while ensuring optimal outcomes. This suggests that, with proper patient selection and management, fast-track protocols can be a viable alternative, achieving outcomes comparable to traditional post-operative care strategies while reducing the burden on intensive care resources.

In our study, the average length of stay was 1.17 days, significantly shorter than the 2.3 days reported in some earlier studies [27].

The incidence of postoperative complications in our study, including surgical revision (2%), minor stroke (0.3%), and acute myocardial infarction managed with angioplasty (0.2%), is notably lower compared to the higher rates reported in the literature following CEA [21]. These findings highlight that the FT protocol does not compromise patient safety, reinforcing its value in reducing postoperative stress.

In the analysis of secondary endpoints, postoperative pain (NRS > 4) was reported by only 0.6% of patients; this may be due to the lingering effects of the deep and superficial cervical blocks, which typically last between 8 to 12 h and 4 to 8 h, respectively, depending on the local anesthetic used for intraoperative infiltration, which is administered at the perivascular level. The high rate of effective pain control contributes to improving patient comfort and satisfaction. We have also found that our protocol is both safe and effective when used in elderly and multimorbid patients. In fact, approximately one-third of our patients were octogenarians, and 40% had an American Society of Anesthesiologists (ASA) score greater than 3.

The social value of a patient’s care cycle can be described as achieving optimal clinical outcomes relative to the associated costs. Efficient utilization of limited hospital resources remains critical for the economic sustainability of the healthcare system. The implementation of the fast-track protocol—characterized by surgical indication based on two DUS exams, the use of LA, eversion CEA, shorter hospital stays, and ICU utilization reserved for only the most critical cases—suggests significant potential for reducing economic burden while maintaining high standards of care.

This study offers a robust dataset for analysis ensuring a focused evaluation of elective carotid endarterectomy.

While the findings provide valuable insights, it is important to acknowledge certain limitations, such as the retrospective nature of the study and the potential biases inherent in a single-center data collection. The absence of a randomized control group represents a significant limitation, which we explicitly acknowledge.

## 5. Conclusions

In conclusion, the promising results of this study support the routine use of this fast-track protocol for elective carotid endarterectomy in asymptomatic patients. By contributing to the evolving landscape of perioperative care, this study encourages further exploration and refinement of fast-track protocols to optimize patient outcomes in vascular surgery. FT protocols for CEA may also enhance cost-effectiveness, strengthening its competitiveness against endovascular interventions by reducing healthcare expenditures. This dual advantage—clinical excellence and economic sustainability—reinforces the role of CEA as a preferred therapeutic approach.

## Figures and Tables

**Table 1:** Main features of carotid surgery fast-track protocol.

Preoperative	
Surgical intervention indication	Two duplex ultrasound examinations, conducted by two different operators.
Hospital admission	The patients are admitted right before surgery.
Blood pressure management.	angiotensin receptor blockers, ACE inhibitors, calcium channel blockers
**Intraoperative**	
Surgical timing	The CEA procedure is performed during the morning surgical session, ensuring at least 6 h of close postoperative monitoring before night shift.
Anesthesia Protocol	Local anesthesia Echo-guided superficial cervical plexus block plus infiltration of local anesthetic along the cutaneous incision line (up to a maximum of ropivacaine 75 mg, lidocaine 200 mg)
Neurological monitoring	Clinical examination (allowing selective shunting only for patients showing neurological impairment) Movement check, time/place orientation assessment, basic cognitive task performance
Patient coagulation management	Heparin before clamping 60–100 U/kg and a target ACT of 200–250 Protamine to reverse half heparin dose if intraoperative check shows no technical defects.
Surgical technique	Eversion
Intraoperative control	Completion angiography
Postoperative latero-cervical drainage	Selective
Dressing	Light wound dressing
**Postoperative**	
Transfer to an intensive care unit	Multimorbid patients or severe intraoperative complications
Postoperative monitoring	At the end of the surgical procedure: close monitoring in the operating recovery room for one hour At readmission to the ward: nursing staff, along with medical personnel, perform a neurological examination to assess any changes compared to the preoperative state. An ECG is performed, and the patient is monitored using telemetry. On postoperative day 0: Vital signs are measured every 3 h, concurrently assessing the trachea alignment and the potential presence of cervical hematoma.
Early postoperative recovery programs	Patients are mobilized 4 h postoperatively, allowed to drink after 2–4 h, and have a light dinner on day 0.
Discharge 1st postoperative day	Yes if no pain, no ECG changes, hemodynamic stability, no neck hematoma, no cranial nerve injury, easy access to hospital readmission

**Table 2:** Demographic characteristics.

**Age (mean)**	74
**Age > 80**	234 (27)
**Sex**	M 544 (64); F 309 (36)
**Arterial Hypertension**	738 (86)
**Dyslipidemia**	698 (82)
**Diabetes Mellitus**	244 (29)
**Smoking (active or former)**	182 (21)
**COPD**	134 (16)
**CKD**	85 (10)
**Ischemic heart disease**	297 (35)
**ASA SCORE (3–4)**	370 (43)

## Data Availability

The original contributions presented in this study are included in the article. Further inquiries can be directed to the corresponding author.

## Abbreviations

The following abbreviations are used in this manuscript:

FT	fast track
CEA	carotid endarterectomy
NRS	numerical rating scale
ICU	intensive care unit
CAS	carotid artery stenting
SAP	systolic arterial pressure
CTA	computed tomography angiography
DUS	duplex ultrasound
LA	local anesthesia
GA	general anesthesia
DAPT	dual antiplatelet therapy
ASA	American Society of Anesthesiologists
ERAS	enhanced recovery after surgery
ESVS	European Society of Vascular Surgery

## Appendix A

### Humanitas University Fast-Track Protocol for Carotid Surgery

**Surgical indication**

Carotid endarterectomy (CEA) is based exclusively on two independent carotid ultrasound examinations performed by different operators. Computed tomography angiography (CTA) is reserved for patients in whom ultrasound findings are inconclusive or difficult to interpret.

**Preoperative assessment**

Includes routine blood tests, a specialist cardiology consultation with an electrocardiogram, and an anesthesiology consultation.

**Hospital admission**

Patients are admitted directly for surgery, either on the afternoon before the procedure (if scheduled as the first case the following morning) or on the morning of surgery.

**Perioperative blood pressure management**

Antihypertensive medications, including ACE inhibitors, angiotensin II receptor blockers (ARBs), and calcium channel blockers, are withheld to avoid intraoperative hypotension on the day of the procedure and allow “permissive” hypertension during the clamping phase. This approach is supported by evidence in the literature, which highlights that the perioperative use of ARBs and ACE inhibitors increases the risk of hypotension during anesthesia. Similarly, taking calcium channel blockers on the day of surgery can complicate intraoperative blood pressure management [28,29].

**Surgical timing**

Procedures are performed in the morning to allow for at least 6 h of strict postoperative monitoring.

**Anesthesia Protocol**

Surgery is performed under local anesthesia using an ultrasound-guided superficial cervical block combined with infiltration along the incision line, with a total of 75 mg ropivacaine and 200 mg lidocaine, plus an additional 20 mg available for administration during vessel isolation, as needed. We routinely infiltrate the carotid sinus with local anesthetic. This technique has been recommended to minimize blood pressure fluctuations during CEA, although its effectiveness remains controversial [30].

**Surgical technique**

The surgical approach for carotid exposure was performed according to the standardized method [31].

Intraoperative cranial nerve monitoring is not routinely employed in our practice. Instead, nerve injury is mitigated using anatomical landmarks and meticulous dissection techniques, in accordance with the ESVS guidelines.

Carotid endarterectomy is usually performed using the eversion technique [32].

**Neurological monitoring**

During carotid clamping, the anesthesia team evaluates the patient’s consciousness, absence of lateralizing neurological deficits, by checking limb movement on the contralateral side, orientation in time and space, and ability to perform basic cognitive tasks.

**Shunting**

Shunting, in our protocol, is used selectively in patients who show neurological impairment during intraoperative monitoring.

**Hemodynamic monitoring**

Relies on non-invasive continuous assessment of arterial pressure and cardiac output with the volume-clamp method [33].

**Anticoagulation strategy**

Heparin before clamping 60–100 U/kg and a target ACT of 200–250.

Surgical technique: carotid endarterectomy (CEA) is performed using eversion as preferred technique

**Intraoperative imaging**

Completion intraoperative angiography is routinely performed via direct puncture of the common carotid artery to assess the plaque endpoint and to confirm the absence of technical defects or residual stenosis. The angiography requires a maximum of 5 mL of contrast medium, making it a feasible option even for patients with mild to moderate renal insufficiency, provided they receive appropriate preoperative hydration.

**Heparin reversal**

Protamine to reverse half heparin dose if the intraoperative check shows no technical defects to ensure effective hemostatic control, facilitating wound closure without the necessity of a drain.

**Drainage strategy**

Drainage is selectively applied to patients on dual antiplatelet therapy or if hemostasis is not achieved within 10 min after declamping.

**Light wound dressing**

Flat, non-compressive wound dressing.

**Selective transfer to an intensive care unit**

Indication limited to severely multimorbid patients or in case of severe intraoperative complications.

**Postoperative monitoring**

Once the patient returns to the ward, the nursing staff and medical team together conduct a comprehensive neurological assessment. This includes evaluating motor function and sensation on the side contralateral to the surgical site, cranial nerve function, monitoring the patient’s level of consciousness, and identifying any potential focal deficits or signs of cerebral hyperperfusion. The findings are compared with the patient’s preoperative neurological baseline to detect any changes or complications. An ECG is performed, and the patient continues to be monitored using telemetry. Vital signs are measured every 3 h on day 0, concurrently assessing the trachea alignment and the potential presence of cervical hematoma.

**Early postoperative recovery programs**

Four hours post-surgery, patients are encouraged to sit and drink. The evening of surgery the patients are mobilized and allowed to eat

**Discharge**

Patients are discharged on the first postoperative day if they are free of pain, show no ECG changes, demonstrate hemodynamic stability, have no neck hematoma or cranial nerve injury, and have easy access to hospital readmission.
